# Neutrophil gelatinase-associated lipocalin and acute kidney injury in endovascular aneurysm repair or open aortic repair: a pilot study

**DOI:** 10.11613/BM.2018.010904

**Published:** 2018-01-10

**Authors:** Benedetta Rampoldi, Serena Tessarolo, Paola Giubbilini, Paola Gaia, Samantha D. Corino, Sarah Mazza, Roberta Rigolini, Marco Dei Poli, Elena Vianello, Massimiliano M. Corsi Romanelli, Elena Costa

**Affiliations:** 1 Laboratory Medicine Operative Unit 1 - Clinical Pathology, I.R.C.C.S. Policlinico San Donato, San Donato Milanese, Milan, Italy; 2 Intensive Care Unit, I.R.C.C.S. Policlinico San Donato, San Donato Milanese, Milan, Italy; 3 Department of Biomedical Sciences for Health, Università degli Studi di Milano, Milan, Italy

**Keywords:** acute kidney injury, neutrophil gelatinase-associated lipocalin, cardiovascular surgery, early detection, urinary biomarkers

## Abstract

**Introduction:**

Acute kidney injury (AKI) occurs frequently after abdominal aortic surgery and there is currently no effective marker able to detect early onset. The aim of this study is to evaluate the ability of neutrophil gelatinase-associated lipocalin (NGAL) to early identify the development of acute renal damage in patients undergoing endovascular aneurysm repair (EVAR) or open aortic repair (OAR).

**Materials and methods:**

Serial samples of blood and urine were obtained from 25 patients undergoing both EVAR and OAR. Seven male subjects with AKI and 18 subjects with no-AKI (17 males, 1 female) were included in the study. We determined concentrations of serum creatinine (sCr) and urinary, serum and whole blood NGAL (uNGAL, sNGAL, bNGAL) collected at baseline, and after 4 and 18 hours. AKI was defined according to the RIFLE criteria (risk, injury, failure, loss of kidney function, and end-stage kidney disease): increase by 50% in sCr or reduction of at least 25% of estimated glomerular filtration rate (eGFR) from baseline.

**Results:**

Seven patients developed AKI in the stage Risk. There was no significant difference in sNGAL concentrations in the AKI group as compared to no-AKI group. However, the uNGAL/uCreatinine ratio and bNGAL concentrations were significantly higher after 18 hours in the AKI group (no-AKI 1.69 (0.91 - 2.47) *vs* AKI 3.2 (2.08 - 5.92) ng/mg for uNGAL/uCreatinine ratio, P = 0.036; and no-AKI 83 (59 - 131) vs AKI 164 (126 – 263) ng/mL for bNGAL, P = 0.029).

**Conclusions:**

Our results suggest that uNGAL, sNGAL and bNGAL, after abdominal aortic surgery, are not suitable as early biomarkers of AKI.

## Introduction

Cardiovascular surgery is considered by the American Heart Association (AHA/ACC) as a major risk procedure for the development of cardiopulmonary and renal complications. In particular, acute renal failure is quite common in patients undergoing endovascular aneurysm repair (EVAR) or open aortic repair (OAR). A transient or permanent reduction of renal function following major vascular surgery is described in a range of between 1 and 28%. In particular, a retrospective study showed a greater incidence of acute kidney injury (AKI) in patients undergoing OAR (26.3%) compared to EVAR (5.5%) (*1*).

Despite the progress made in recent years in understanding acute renal failure pathogenesis, many therapeutic interventions have proved ineffective (*2*). This failure is partly determined by the lack of a sensitive and specific renal marker that allows an early diagnosis and therefore a timely treatment of the disease.

Recently, scientists have focussed on neutrophil gelatinase-associated lipocalin (NGAL), a 25 kDa glycoprotein of the lipocalin superfamily. It is normally expressed in low concentrations in various human tissues and it is induced in the kidney early after ischemic or nephrotoxic damage. Neutrophil gelatinase-associated lipocalin has proven to be an early marker of AKI in paediatric and adult patients undergoing cardiac surgery (*3*).

Assuming that there is an increase in NGAL at 4 hours from baseline, the aim of this pilot study is to evaluate the ability of NGAL protein, measured in serum, whole blood and urine samples, to early identify the development of acute renal damage in patients undergoing EVAR or OAR. The choice of these matrices is dictated by the need to compare the reliability of a point of care with respect to other commercially available techniques.

## Materials and methods

### Subjects

A cross-sectional study of diagnostic accuracy was conducted at IRCCS Policlinico San Donato. During the study period (October 2015 - October 2016), 25 patients (24 male and 1 female) were hospitalized in the Vascular Surgery Department for EVAR or OAR. They were monitored during the postoperative period for possible onset of AKI defined according to the RIFLE criteria (risk, injury, failure, loss of kidney function, and end-stage kidney disease). The latter is based on increased serum creatinine (sCr) by 1.5 times or decreased estimated glomerular filtration rate (eGFR) by at least 25% with respect to the baseline values (*4*). Subjects who did not develop AKI (N = 18) were included in the no-AKI group while subjects who reported a serum creatinine increase by 1.5 times or an eGFR 25% lower than baseline were included in the AKI group (N = 7). NGAL protein measurements were performed and compared with traditional renal function markers, *i.e.* sCr and eGFR calculated using the Modification of Diet in Renal Disease study equation (MDRD) formula (*5*). For each subject, whole blood, serum and urine samples were collected in three phases defined by the protocol. The baseline sample was the blood and urine sampling performed prior to surgery, while the subsequent sampling was performed at 4 and 18 hours from baseline, which is the time of abdominal clamping in the case of traditional intervention or the last administration of contrast media in the case of endovascular exclusion.

Urine sample was used for creatinine assay and then, after centrifugation at 400xg for 5 minutes, the supernatant was separated in 10 mL aliquots and stored at - 80 °C for the NGAL assay. Serum sample, obtained after centrifugation at 1000xg for 10 minutes, was used for the creatinine assay and then it was stored at - 80 °C until NGAL assayed. Whole blood sample was used immediately for NGAL assay by a rapid-point-of-care fluorescence immunoassay. Serum sample was collected in plastic micronized silica particles tube (4 mL, 13x75 mm, ref number 368813) and whole blood sample was collected in a tube (2 mL, 13x75 mm, ref number 367836) with tripotassium salt of ethylenediaminetetraacetic acid (K_3_EDTA) as anticoagulant (Becton, Dickinson and Company, Franklin Lakes, USA).

The study protocol was approved by “Comitato Etico Indipendente (C.E.I.) dell’Azienda Socio Sanitaria Territoriale (A.S.S.T.) Melegnano e della Martesana”. All subjects signed the informed consent and each procedure was conducted in accordance with the 1964 Helsinki Declaration and subsequent amendments (last amendment October 2013). For both groups, inclusion criteria were abdominal aortic aneurysm, age > 18 years and signed informed consent. Exclusion criteria were the extension of the chorea aura aneurysm, pre-existing chronic renal failure defined by the National Kidney Foundation/Kidney Disease Outcomes Quality Initiative (NKF/KDOQI) guidelines and age > 80 years.

### Methods

Serum creatinine (intra-assay coefficient of variation (CV) = 1.4%) and urine creatinine (intra-assay CV = 1.4%) were measured using Roche Cobas C501 platform (Roche Diagnostics GmbH, Mannheim, Germany). Serum creatinine assay is standardized by tracing reference materials to Isotope Dilution Mass Spectrometry (IDMS). Urinary NGAL (uNGAL) concentrations were determined by Abbott ARCHITECT i1000SR® analyzer (Abbott Diagnostics GmbH, Wiesbaden, Germany) using an immunological chemiluminescence microparticle capture (CMIA) assay (intra-assay CV = 3.2%).

Concentrations of NGAL in whole blood samples (bNGAL) were determined by Triage® NGAL Test, a point-of-care rapid fluorescence immunoassay performed on a Triage Meter analyser (Biosite Incorporated, San Diego, California) (intra-assay CV = 12.8%). Serum NGAL (sNGAL) concentrations were determined by a Quantikine® Enzyme-Linked Immunosorbent Assay (ELISA) Human Lipocalin-2/NGAL Immunoassay (R&D Systems a Bio-techne brand, Inc., Minneapolis, USA) using a direct immunoenzymatic assay (ELISA sandwich) (intra-assay CV = 3.7%). All CVs are those declared by manufacturers.

### Statistical analysis

The results are reported as median and interquartile range (IQR) for continuous variables. Mann-Whitney U-test was used to compare continuous variables between the two groups observed. The discriminating capacity of markers was assessed by building the Receiver Operating Characteristic (ROC) curve. A P-value of < 0.05 was considered statistically significant. All statistical analyses were performed using the SPSS Statistics program, version 21.0 (IBM, Chicago, USA).

## Results

**Figure 1:**
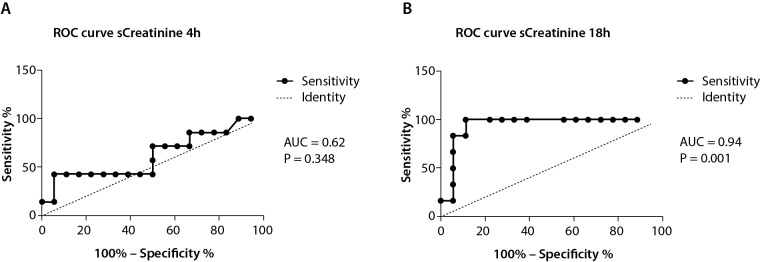
ROC curves for sCreatinine. Panel A: sCreatinine at 4 h did not discriminate between patients without renal disease following surgery compared to those with AKI (AUC (95% CI) = 0.62 (0.36 - 0.89); P = 0.348). Panel B: sCreatinine performed at 18 h shows that it is possible to separate the two patient groups (AUC (95% CI) = 0.94 (0.85 - 1.04); P = 0.001). sCreatinine - serum creatinine. AKI - acute kidney injury. AUC - area under curve.

**Figure 2:**
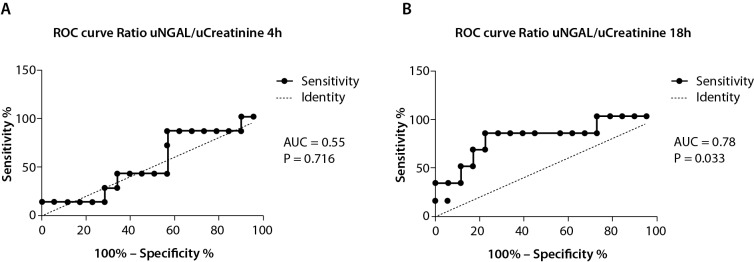
ROC curves for uNGAL/uCreatinine ratio. Panel A: uNGAL/uCreatinine ratio at 4 h did not discriminate between patients without renal disease following surgery compared to those with AKI (AUC (95% CI) = 0.55 (0.30 - 0.80); P = 0.716). Panel B: uNGAL/uCreatinine ratio performed at 18 h shows that it is possible to separate the two patient groups (AUC (95% CI) = 0.80 (0.57 - 1.02); P = 0.033). uCreatinine - urinary creatinine. AKI - acute kidney injury. uNGAL - urinary neutrophil gelatinase-associated lipocalin. AUC - area under curve.

**Figure 3:**
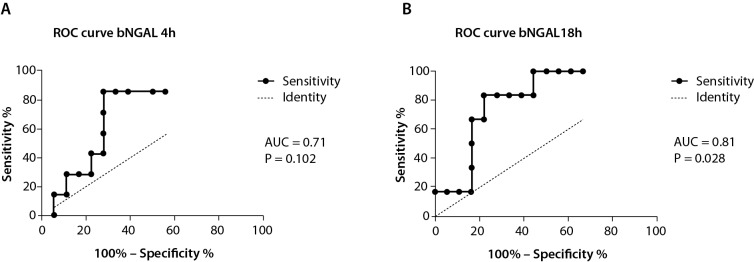
ROC curves for bNGAL. Panel A: bNGAL at 4 h did not discriminate between patients without renal disease following surgery compared to those with AKI (AUC (95% CI) = 0.71 (0.49 - 0.94); P = 0.102). Panel B: bNGAL performed at 18 h shows that it is possible to separate the two patient groups (AUC (95% CI) = 0.81(0.63 - 0.99); P = 0.028). bNGAL - whole blood neutrophil gelatinase-associated lipocalin. AKI - acute kidney injury. AUC - area under curve.

A total of 25 patients were enrolled in the study. According to the RIFLE criteria, 7 developed AKI within the Risk category while 18 subjects were included in the no-AKI group. Serum creatinine concentrations both prior to intervention and at 4 hours showed no significant differences between no-AKI and AKI patients (at baseline: no-AKI = 72 (64 – 84) *vs* AKI = 90 (72 – 95) µmol/L; P = 0.115; at 4h: no-AKI = 72 (59 – 85) *vs* AKI = 74 (67 – 91) µmol/L; P = 0.364). On the contrary, at 18 hours sCr concentrations were significantly higher in the AKI group with respect to no-AKI group (114 (100 – 130) µmol/L and 82 (59 – 87), P = 0.001, respectively). The results of uNGAL, sNGAL and bNGAL assays are summarized in Table 1. A ROC curve analysis was performed to demonstrate the capability of the diagnostic test in discriminating patients who developed AKI after EVAR or OAR. The results of the ROC curve for serum creatinine, ratio uNGAL/uCreatinine and bNGAL are described in Figures 1-3. Serum NGAL ROC curve shows that the NGAL marker on serum samples is unable to discriminate between AKI and no-AKI patients.

**Table 1:** Concentrations of uNGAL/uCr ratio, bNGAL and sNGAL at different time points

	**No-AKI** **(N = 18)**	**AKI** **(N = 7)**	**P**
**uNGAL/uCr ratio (ng/mg)**			
baseline	1.18 (0.69 – 3.17)	0.84 (0.61 - 1.31)	0.380
4h	2.25 (1.14 – 4.55)	1.97 (1,89 - 4.12)	0.739
18h	1.69 (0.91 - 2.47)	3.20 (2.08 - 5.92)	0.035
**bNGAL (ng/dL)**			
baseline	59 (59 - 74)	60 (59 – 118)	0.580
4h	64 (59 – 105)	102 (82 – 144)	0.101
18h	83 (59 – 131)	164 (127 - 201)	0.029
**sNGAL (ng/dL)**			
baseline	138 (64 – 188)	125 (80 – 161)	0.931
4h	174 (85 - 215)	173 (140 – 226)	0.714
18h	137 (98 – 217)	185 (153 – 226)	0.429
The results are expressed as median and IQR. P < 0.05 was considered statistically significant. NGAL - neutrophil gelatinase-associated lipocalin. uNGAL - urinary NGAL. bNGAL - whole blood NGAL. sNGAL - serum NGAL. uCr - urinary creatinine. AKI - acute kidney injury.			

## Discussion

From the analysis of the results we can suggest that the NGAL protein in the three different matrices studied is not an early biomarker of AKI. The 18-hour sCr assay showed a statistically significant increase in the AKI group although we would have expected a significant increase not earlier than 24 hours from baseline (*6*). The statistical analysis of sNGAL assays by ELISA did not show statistically significant differences between AKI and no-AKI group. At 18 hours we observed statistically significant difference in uNGAL/uCreatinine ratio and in bNGAL concentrations between the two groups but we would have expected a significant increase already at 4 hours, which did not occur.

According to our knowledge there are not many studies that have examined the role of NGAL in predicting the development of AKI in similar patients’ population. In 2017 Noorani *et al.* assessed changes in urinary biomarkers, including NGAL, interleukin 18 (IL-18) and sCr in patients undergoing EVAR. A significant increase in NGAL and IL-18 was observed at 6 hours after the procedure while sCr concentration rose only after 24 hours (*7*). Brinkman *et al.* investigated the ability of NGAL in predicting AKI in patient undergoing OAR. They demonstrated that postoperative urinary NGAL has the ability to predict the development of a subsequent AKI (*8*). In recent years, other emerging urinary biomarkers have also been considered to identify early onset of AKI in subjects undergoing EVAR and OAR. In particular, in 2014 Pirgakis *et al.* have assessed the ability of urinary cystatin C (uCysC) to detect renal dysfunction earlier than serum creatinine. It has been observed that in the AKI group the urinary cystatin C concentrations at 6 hours were significantly higher than no-AKI patients (*9*). Another promising candidate as kidney injury biomarker is the liver-type fatty-acid-binding protein (L-FABP). In 2016 Obata *et al.* described urinary L-FABP as an early AKI marker in the same patient population. In fact, a significant increase in urinary L-FABP in the AKI group was observed at 4 h for patients undergoing EVAR and at 2 h after aortic cross-clamping (AXC) for patients undergoing OAR (*10*).

In our study ROC curve analysis for different biological matrices suggests that urine and whole blood NGAL assay can be a good diagnostic test at 18-hour from time 0 but it is not an early biomarker of AKI. The low number of analysed samples limits the power of the analysis and does not allow for a cut-off value in the diagnosis of AKI.

Our study presents some limitations. First limitation is its small sample size, although the duration of our recruitment was 12 months, so we want to recruit a larger sample sizes to confirm our findings. Second, AKI is detected according to RIFLE criteria that represent an imperfect estimate of the kidney function and we do not consider other surrogates like survival, intra-hospital complications and decline of kidney function in long-term. However, we think the study is interesting because there are not many papers in the literature that take into account similar patients.

In conclusion, contrary to the principal findings in literature, our data do not allow us to state that NGAL is an early marker of AKI in patients undergoing EVAR and OAR.
